# Intravitreal dexamethasone implants for the treatment of refractory scleritis combined with uveitis in adult-onset Still’s disease: a case report

**DOI:** 10.1186/s12886-016-0380-4

**Published:** 2016-11-08

**Authors:** Seong Joon Ahn, Sun Jin Hwang, Byung Ro Lee

**Affiliations:** Department of Ophthalmology, Hanyang University Hospital, Hanyang University College of Medicine, 17 Haengdang-dong, Seongdong-gu, Seoul 133-792 South Korea

**Keywords:** Adult-onset Still’s disease, Case report, Dexamethasone implants, Intravitreal injection, Scleritis, Uveitis

## Abstract

**Background:**

Adult-onset Still’s disease is a systemic inflammatory disease which presents with uveitis and scleritis in the eye. Intravitreal dexamethasone implants are used for the treatment of refractory uveitis.

**Case presentation:**

A 19-year-old woman diagnosed to have adult-onset Still’s disease for fevers, joint pain, and a salmon-colored bumpy rash presented with scleritis and uveitis in the left eye. Topical and systemic steroids with oral methotrexate failed to control the inflammation. We performed intravitreal injections of dexamethasone implants for side effects of steroid and refractory ocular inflammation. The therapy resulted in improvements in the patient’s uveitis with reductions in scleral vessel engorgement and redness. There was no recurrence of uveitis or scleritis during 4 months following treatment.

**Conclusions:**

Intravitreal injections of dexamethasone implants may result in clinical improvements of refractory scleritis combined with uveitis.

## Background

Scleritis is an uncommon but severe, painful, and potentially blinding ocular disease characterized by chronic inflammation of the sclera that is occasionally accompanied by uveitis [1]. Although topical or systemic corticosteroids are typically used for treatment, sometimes combined with systemic immunosuppressive agents or biologics, some cases have chronic (persistent) or recurrent inflammation [2–4], which requires other therapeutic modalities.

Dexamethasone implants (Ozurdex®, Allergan, USA) are intravitreal, sustained release corticosteroid implants. Such implants are approved for the treatment of macular edema secondary to diabetic retinopathy [5] or retinal vein occlusion [6] and non-infectious posterior uveitis [7]. The implant slowly releases dexamethasone for up to 6 months, which makes it effective for the treatment of frequently recurring diseases such as macular edema and uveitis, as it may reduce the number of intravitreal injections required.

In this report, we describe a case of adult-onset Still’s disease with refractory scleritis and uveitis that was successfully treated with intravitreal dexamethasone implants.

## Case presentation

A 19-year old female visited our clinic for uncontrolled scleritis in the left eye. She denied any previous intraocular inflammation, ocular injury, or surgery but she had been diagnosed with adult-onset Still’s disease by a rheumatologist due to persistent fevers, joint pain, and a salmon-colored bumpy rash, for which systemic steroid and immunosuppressive agents had been prescribed. Before her visit to our clinic, she had suffered from recurrent episodes of scleritis.

**Fig. 1:**
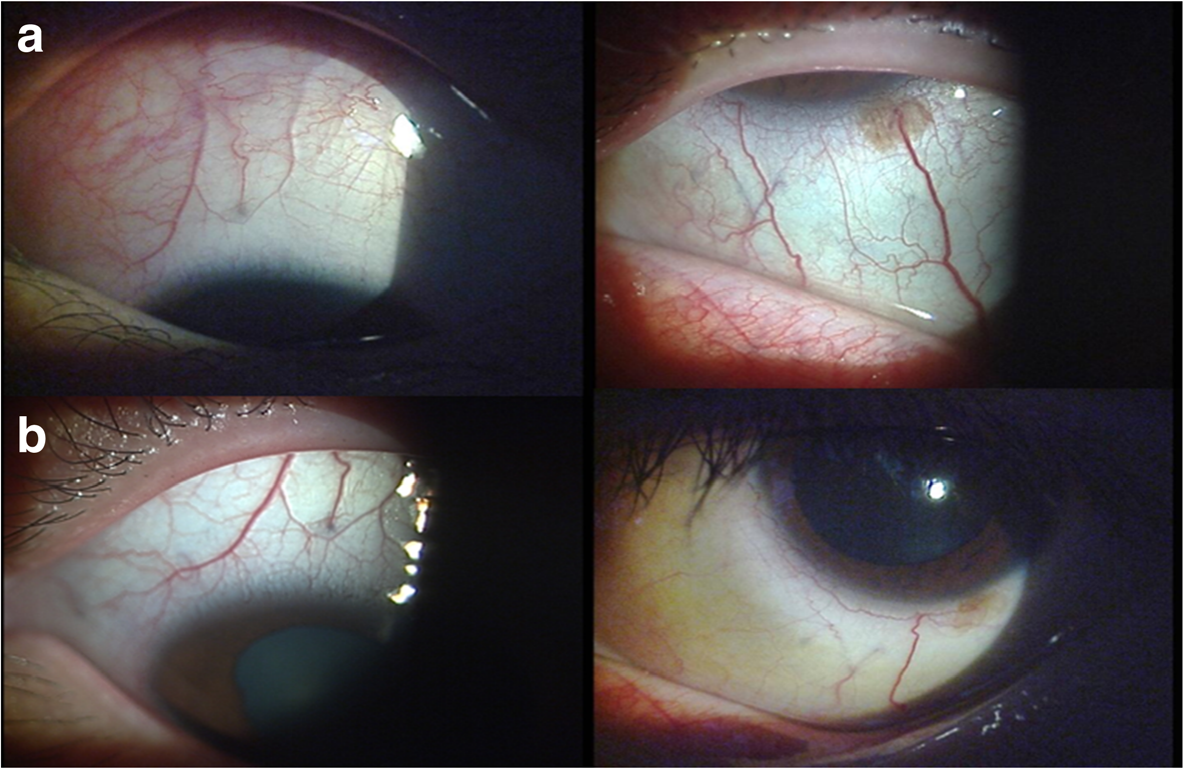
Anterior segment photograph of sectoral scleritis before **a** and after **b** intravitreal dexamethasone implant injection. **a** Despite the long-term use of topical and systemic steroids, anterior segment photographs reveal scleral vessel engorgement and redness in the superonasal sclera of the left eye. **b** At 1 month after intravitreal dexamethasone implant injection, the redness and engorgement of the scleral vessels improved. Combined uveitis was resolved following the injection

**Fig. 2:**
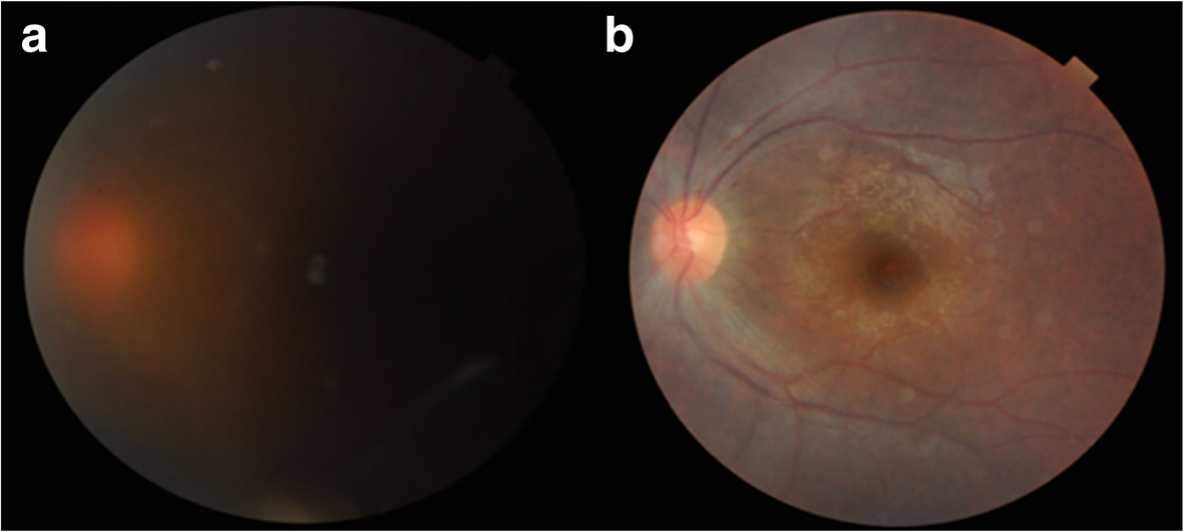
A fundus photograph of the left eye showing a hazy posterior pole due to vitritis **a**. The fundus photo obtained at 1 month after intravitreal dexamethasone implant injection **b** shows a much clearer fundus view and normal posterior pole

Her vision was 20/20 OD and 20/30 OS at the first visit. Slit-lamp examination revealed scleral vessel engorgement and redness in the superonasal sclera of the left eye, which was compatible with sectoral scleritis. The eye also showed inflammation in the anterior chamber and vitreous, to the degree of grade 2+ according to the Standardization of Uveitis Nomenclature (SUN) Working Group [8]. Other than posterior synechia, there were no other abnormal findings in the anterior segment. Fundus examination of both eyes revealed a normal posterior pole and retinal periphery. She received subtenon triamcinolone injections, topical 1 % prednisolone acetate four times a day, oral prednisolone 30 mg / day (0.5 mg / kg), and methotrexate 15 mg per week to control the intraocular and scleral inflammation. After 12 weeks, despite the systemic therapy, slit-lamp examination revealed persistent scleritis (Fig. 1a) and vitritis in the left eye (Fig. 2a). She complained of weight gain and moon face. However, as articular and other systemic symptoms had been well controlled by the medication, local therapy with dexamethasone implant was considered before the systemic use of biologic agents such as TNF-alpha antagonists.

Accordingly, we performed intravitreal injection of a dexamethasone 0.7-mg implant (Ozurdex; Allergan, Irvine, CA) into the vitreous of the left eye to treat the patient’s uveitis refractory to topical and systemic treatment. Two weeks later, she informed us that her vision and redness in the left eye were improved, and her scleral vessel engorgement and redness were almost resolved. One month following the injection, the intraocular inflammation was improved from cell 2+ to cell trace in the anterior chamber and from cell 2+ to none in the vitreous and the scleritis was almost completely resolved (Figs. 1b and 2b). At the last visit, 4 months after the dexamethasone implant injection, there had been no recurrence of scleritis or uveitis although systemic corticosteroids had been tapered and discontinued. There were no drug-related side effects such as increased intraocular pressure or cataract progression during the follow-up period. At the final visit, the patient’s vision was 20/22 OD and 20/30 OS.

## Conclusions

Adult-onset Still’s disease is a rare systemic inflammatory disease of unknown etiology characterized by the classic triad of persistent high spiking fevers, joint pain, and a distinctive salmon-colored bumpy rash [9, 10]. Although prognosis is usually favorable, complications affecting the lungs, heart, or kidney can be life-threatening [11]. Scleritis and uveitis are severe intraocular complications occurring in patients with Still’s disease [12]. In this report, we successfully treated scleritis and uveitis in a patient with adult-onset Still’s disease that was resistant to remission and characterized by frequent recurrence with conventional systemic corticosteroids and immunosuppressants via intravitreal dexamethasone implants.

Intravitreal dexamethasone implants are a widely used and safe treatment for uveitis [13]. In particular, such treatment can minimize systemic drug-related complications caused by systemic steroids, immunosuppressive drugs, or biologicals, as it is delivered locally into the eye. Considering the young age of the patient, systemic adverse effects of long-term systemic corticosteroid use, and frequent recurrence of uveitis, intravitreal injections of dexamethasone implants were used rather than dose escalation of systemic steroids or immunosuppressants, as the patient’s main manifestation of disease was ocular inflammation. Unexpectedly, the patient’s refractory scleritis also resolved following intravitreal dexamethasone implant injection, suggesting the therapeutic effectiveness of the therapy for scleritis.

In terms of safety, local therapy with dexamethasone implants may be a good treatment option for scleritis, with reduced risk of systemic adverse effects. A previous report showed that subconjunctival dexamethasone implants may be effective and safe as a local therapy for non-necrotizing scleritis [14]. However, subconjunctival administration of depot corticosteroids is considered unsafe owing to the risk of scleral thinning and perforation [15, 16]. Although cataract progression, increased intraocular pressure, and complications associated with intravitreal injections should be considered carefully, intravitreal dexamethasone implants may be an attractive choice for the treatment of scleritis, particularly if uveitis is coexistent.

In summary, we report a case of adult-onset Still’s disease with combined scleritis and uveitis refractory to conventional topical/systemic steroids and immunosuppressants, which resolved after intravitreal dexamethasone implant injections. To our best knowledge, this is the first report describing the therapeutic effect of intravitreal dexamethasone implants for scleritis. Future studies with larger samples to validate the safety and efficacy of the therapy for cases with scleritis with or without uveitis are warranted.

## Abbreviation

SUN: Standardization of Uveitis Nomenclature.
